# A simpler primate brain: the visual system of the marmoset monkey

**DOI:** 10.3389/fncir.2014.00096

**Published:** 2014-08-08

**Authors:** Samuel G. Solomon, Marcello G. P. Rosa

**Affiliations:** ^1^Department of Experimental Psychology, University College LondonLondon, UK; ^2^Department of Physiology, Monash University, Clayton, VICAustralia; ^3^Monash Vision Group, Monash University, Clayton, VICAustralia; ^4^Australian Research Council Centre of Excellence for Integrative Brain Function, Monash University Node, Clayton, VICAustralia

**Keywords:** vision, retina, thalamus, striate cortex, extrastriate cortex, Callitrichidae

## Abstract

Humans are diurnal primates with high visual acuity at the center of gaze. Although primates share many similarities in the organization of their visual centers with other mammals, and even other species of vertebrates, their visual pathways also show unique features, particularly with respect to the organization of the cerebral cortex. Therefore, in order to understand some aspects of human visual function, we need to study non-human primate brains. Which species is the most appropriate model? Macaque monkeys, the most widely used non-human primates, are not an optimal choice in many practical respects. For example, much of the macaque cerebral cortex is buried within sulci, and is therefore inaccessible to many imaging techniques, and the postnatal development and lifespan of macaques are prohibitively long for many studies of brain maturation, plasticity, and aging. In these and several other respects the marmoset, a small New World monkey, represents a more appropriate choice. Here we review the visual pathways of the marmoset, highlighting recent work that brings these advantages into focus, and identify where additional work needs to be done to link marmoset brain organization to that of macaques and humans. We will argue that the marmoset monkey provides a good subject for studies of a complex visual system, which will likely allow an important bridge linking experiments in animal models to humans.

## INTRODUCTION

Despite advances in non-invasive techniques for study of the living human brain, animal studies are still a necessary approach for understanding the nervous system. Many of the biochemical and physiological operations carried out by neurons represent common, fundamental functions that need to be carried out by all nervous systems. Moreover, the basic anatomical plan of organization of the mammalian nervous system is constrained by a common set of developmental mechanisms, which lead to a similar set of subdivisions and interconnections among adults of different species (Krubitzer, 2007). For these reasons non-primate animal models are often appropriate for addressing scientific questions that cannot be explored in humans. Yet, while it is important to recognize the fundamental similarity of nervous systems in general, and mammalian brains in particular, there are also clear variations, which often translate into marked differences in sensory, motor, and cognitive capacities (e.g., Padberg et al., 2007; Buckner and Krienen, 2013; Chaplin et al., 2013b; Fjell et al., 2014).

The visual system is a case in point. The evolution of human societies has been linked to the emergence of a sophisticated visual system, which we share with other primates. For most of the evolution of humans as a species, the capacity to see the world in sharp, colorful, three-dimensional detail, to understand, differentiate, and remember objects in complex contexts, and to use vision to guide skilful behavior have been important to survival. Whereas other animals have eyes that afford higher acuity (e.g., Fox et al., 1976; Reymond, 1987) or more complex color vision (Marshall and Oberwinkler, 1999; Sabbah et al., 2010), it is the balance between evolution of the eye and brain, including in many cases specific anatomical characteristics, that sets primates apart from other groups of animals, including members of other mammalian orders. Thus, research on non-human primates remains, in many cases, the only way to gain insight to many neural systems that are of particular importance to human cognition and health.

The most widely used non-human primate models in neuroscience research, including the visual system, are the various species of the genus *Macaca* (macaque monkeys; for discussion, see Rosa and Tweedale, 2005; Manger et al., 2008). However, the macaque is not always the best model for investigating the primate visual system. As we will argue below, these limitations become particularly obvious when one considers emerging technologies for physiological and developmental studies of the visual system. We propose that the marmoset monkey (*Callithrix* spp.) offers distinct advantages in many contexts, which allow new avenues of investigation of visual anatomy and function. Although no single species is likely to represent the “ideal” model for every scientific question, the marmoset can provide a powerful counterpart to macaque for understanding brain systems that are sufficiently derived, in evolutionary terms, to demand investigation in a primate.

Here we describe the current state of knowledge of the organization of the marmoset visual system, from the retina to the cortex. In order to make this review tractable, we will generally only include references to the work done in marmosets; comparative references can be found within those primary sources. Many features of the marmoset visual system are shared with macaques and humans, and we will not repeatedly highlight those similarities. When applicable, we will note differences, particularly those that may be important in experimental design. We will demonstrate that, unlike as recently as 20 years ago, there is now a substantial body of knowledge on the visual system of the marmoset, which provides a strong foundation for future work.

## THE MARMOSET BRAIN

In general, the term “marmoset” refers to over 20 species of South American monkeys of the family Callitrichidae, which are characterized by small body size, agile movements, and the presence of claw-like nails on the hands and feet. By far the most commonly used species in laboratory studies is the common marmoset (*Callithrix jacchus*); in this review the term “marmoset” will refer to this species. Marmosets naturally live in family groups of 10–15 individuals, are day-active, and inhabit the upper canopy of forested areas, although they are highly adaptable and can be found in urban fringe areas. The adult body size rarely exceeds 20 cm (excluding the long, non-prehensile tail), and body weight is approximately 300 g (Stevenson and Rylands, 1988). Gestation is approximately 5 months, and breeding females generally give birth twice a year, most frequently to non-identical twins. Sexual maturity is reached around 18 months, and the average life span in captivity is about 13 years (Chandolia et al., 2006; Nishijima et al., 2012). Marmosets remain in their social group until adulthood and are cooperative in caring for their offspring.

**Figure 1:**
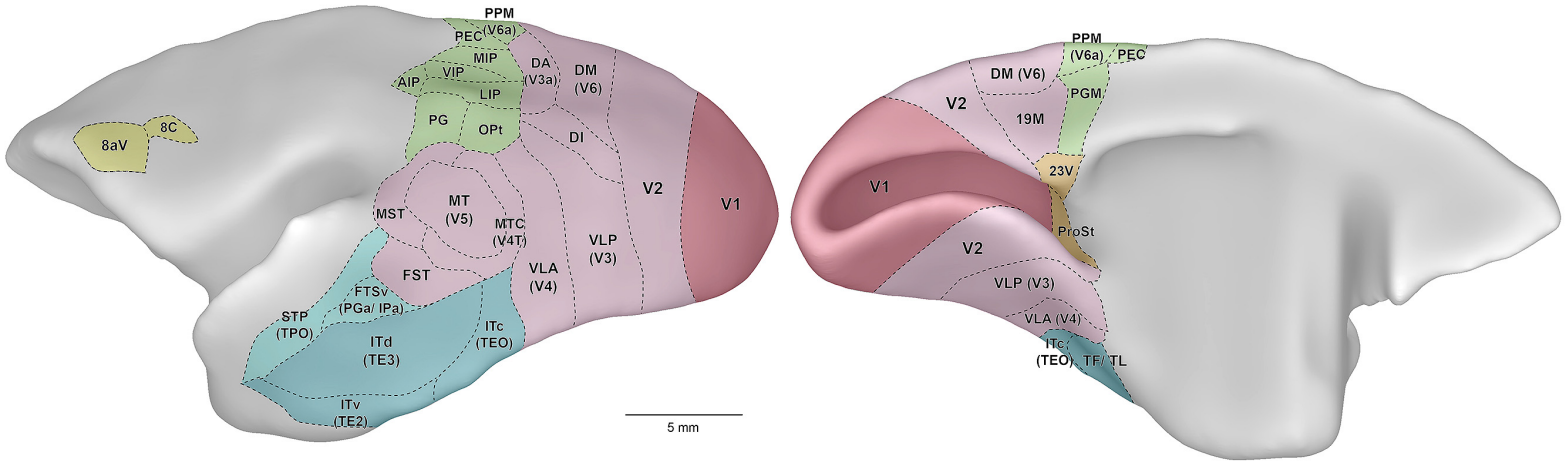
**Lateral (left) and medial (right) views of the marmoset cerebral cortex, showing the location of visual areas.** The images are representations of the reference brain reconstructed in detail by Paxinos et al. (2012). Names within parentheses indicate the names of likely homologous areas in macaque brain. Colors denote different subdivisions of visual cortical pathways, as follows. Magenta: primary visual cortical area (V1). Pink: visuotopically organized areas of extrastriate cortex. Green: posterior parietal cortex. Dark blue: inferior temporal cortex. Light blue: polysensory areas of the superior temporal cortex. Orange: “limbic” visual areas. Yellow: frontal cortex visual association areas, including frontal eye fields. Abbreviations: 8aV, cytoarchitectural area 8a ventral; 23V, cytoarchitectural area 23 ventral; AIP, anterior intraparietal area; DA, dorsoanterior area (probable homolog of macaque area V3a); DI, dorsointermediate area; DM, dorsomedial area (probable homolog of macaque area V6); FST, fundus of superior temporal area; FSTv, fundus of superior temporal ventral area (probable homolog of macaque cytoarchitectural areas PGa and IPa); ITc, caudal inferior temporal area (probable homolog of macaque area TEO); ITd, dorsal inferior temporal area; ITv, ventral inferior temporal area; LIP, lateral intraparietal area; MIP, medial intraparietal area; MST, medial superior temporal area; MT, middle temporal area (probable homolog of macaque area V5); MTC, middle temporal crescent (probable homolog of macaque area V4T); OPt, cytoarchitectural area OPt; PEC, cytoarchitectural area PE caudal; PG, cytoarchitectural area PG; PGM, cytoarchitectural area PG medial; PPM, posterior parietal medial area (probable homolog of macaque area V6a); ProSt, area prostriata; STP, superior temporal polysensory area (probable homolog of macaque cytoarchitectural area TPO); TF/ TL, cytoarchitectural areas TF and TL; V1, primary visual area; V2, second visual area; VIP, ventral intraparietal area; VLA, ventrolateral anterior area (probable homolog of macaque area V4); VLP, ventrolateral posterior area (probable homolog of macaque area V3).

**Figure 1** illustrates the external morphology of the marmoset brain, with visual and visual association cortical areas highlighted. The marmoset brain (∼8 g) is approximately 12 times smaller in volume than that of the rhesus macaque, and 180 times smaller than the human brain (Stephan et al., 1981). **Figure 1** readily conveys one of the key advantages of the marmoset as a model for studies of the visual system: the relatively smooth topology of the cerebral cortex. Thus, in marmosets the vast majority of the visual cortex lies exposed on the surface of the cerebral hemispheres. The only known exceptions are those portions of visual cortex buried in the banks of the calcarine sulcus: that is, the representation of the peripheral visual field in the primary visual cortex (V1; Fritsches and Rosa, 1996), small sectors of the peripheral representation in the second visual area (V2; Rosa et al., 1997), and area prostriata (Yu et al., 2012).

## THE MARMOSET EYE

### OPTICS AND PHOTORECEPTOR DISTRIBUTION

The marmoset eye is large compared to its body weight and brain size, with a diameter of about 11 mm. For details, we direct the reader to the fine schematic marmoset eye provided by Troilo et al. (1993). The size of the marmoset eye is such that near the fovea the retina samples the image with a resolution of about 128 μm/degree. The major distinguishing feature of the primate retina, the *fovea centralis*, appears morphologically similar in the marmoset and Old World monkeys. Cone photoreceptors are small and packed at high density, rod photoreceptors and blood vessels are absent, and the post-receptoral elements are displaced across the retina by up to 1 mm from the photoreceptors (Wilder et al., 1996). The combination of cone density and optical clarity means that potential visual acuity is much higher at the fovea than anywhere else. Cone density reaches approximately 200,000 cones/mm^2^ in the marmoset fovea (Troilo et al., 1993; Wilder et al., 1996; see also Finlay et al., 2008), similar to the peak cone density in macaques and humans (Curcio et al., 1987). The spatial resolution of the photoreceptor mosaic in the marmoset is therefore estimated to be close to 30 cycles/degree, which is near the spatial acuity found in behavioral measurements (Ordy and Samorajski, 1968). Rod photoreceptors are effectively absent from the fovea – they rise to a peak density of approximately 70,000 rods/mm^2^, at about 15° from the fovea (Goodchild et al., 1996; Wilder et al., 1996). The absolute size of the rod-free foveal zone is similar in marmosets and larger primates (Franco et al., 2000; Finlay et al., 2008), and the ratio of cones to rods in peripheral marmoset retina is higher than that in macaque and human retina (Wilder et al., 1996), so marmoset vision may be cone-dominated over a larger fraction of the visual field. Functional correlates of these species differences are yet to be established.

The relatively short gestation time of marmosets makes it easier to study the developing eye and retina, including the emergence of an avascular zone at the fovea and the associated changes in neural organization (Hendrickson et al., 2006, 2009; Springer et al., 2011). The fovea emerges relatively late in marmoset development, but develops rapidly (Hendrickson et al., 2006, 2009). Recent adaptive optics measurements (Coletta et al., 2010) confirm that marmosets are generally hyperopic in early life and become myopic with age. The rapid postnatal maturation of marmosets makes them useful in understanding the neural changes that accompany developmental disorders, including myopia (Troilo and Judge, 1993; Nickla et al., 2002; Troilo et al., 2007), retrograde degeneration triggered by lesions of the visual pathway (Hendrickson et al., 2013), normal aging (Böhm et al., 2013), and potentially diseases related to primate retinal specialization, such as foveal detachment and macular degeneration.

### CONE PHOTORECEPTOR CLASSES

The spectral sensitivity of a photoreceptor is defined by the type of opsin that it expresses, and primate cone photoreceptors can be divided into two classes – those most sensitive to shorter (“blue”) wavelengths, and those most sensitive to longer (“red,” “green”) wavelengths (reviewed by Jacobs, 2008). Shorter wavelengths are subject to greater scatter by the atmosphere and optics, and are not focused at the same point as longer wavelengths, making them less useful for fine spatial vision. Cones most sensitive to short wavelengths (S-cones; “blue”; peak wavelength 423 nm) are relatively rare (5–10% of all cones), are smaller than other cones (Martin and Grünert, 1999), and show some molecular similarities to rods (Craft et al., 2014). These S-cone photoreceptors appear more irregularly distributed in the marmoset (Martin et al., 2000) than in macaque and other Old World monkeys and apes, and are present (at low density) at the center of the fovea (Martin and Grünert, 1999; Hendrickson et al., 2009); some other quantitative aspects of S-cone distribution may also differ from those in the macaque and human retina (Curcio et al., 1991).

In primates the opsins associated with sensitivity to medium-long wavelengths are encoded on the X-chromosome. In macaques and humans, the genes for opsins most sensitive to long (L-cones; “red”) and medium (M-cones; “green”) wavelengths lie in sequence, and a locus control region controls which opsin is expressed in an individual photoreceptor. In marmosets and several other New World monkey species there is instead a single locus, where distinct opsins are encoded as allelic variants (Jacobs, 2008). In the marmoset three alleles code opsins that are most sensitive to 543, 556, or 563 nm (Travis et al., 1988; Tovée et al., 1992; Williams et al., 1992; Hunt et al., 1993; Shyue et al., 1995); which opsin is expressed in females is dictated by inactivation of one of the X-chromosomes early in development. The result is that male marmosets are dichromatic (“red–green color blind”), because the longer wavelength photoreceptors all have the same peak sensitivity. Those female marmosets carrying two distinct alleles are trichromatic, with color vision that depends on the particular combination of opsins present. There is a good match between the capacity for color vision as predicted from opsin genotype and that observed behaviorally: in particular, trichromatic females show behavioral color vision consistent with presence of cone-opponent mechanisms in red–green region of the visible spectrum (Tovée et al., 1992; for similar behavioral work in marmosets other than *C. jacchus*, see also Pessoa et al., 2005; Caine et al., 2010). At mesopic luminances both rods and cones are active, providing a potential source of “trichromacy” in dichromatic marmosets, and there is some evidence that dichromatic marmosets can exploit this potential source of chromatic information (Freitag and Pessoa, 2012).

The polymorphic variation of red–green color vision in marmosets forms a natural model for understanding the impact of red–green color blindness on subsequent visual processing (Jacobs, 2008). As yet no anatomical correlates of color blindness have been found in the retina (Chan and Grünert, 1998; Chan et al., 2001; Jusuf et al., 2006a,b), thalamus, or primary visual cortex (Goodchild and Martin, 1998; Solomon, 2002). The presence of large numbers of dichromatic individuals should also make it possible to ask whether the introduction of novel photoreceptor opsins can be exploited by plasticity in subsequent neural representations, which may directly or indirectly model future treatments of photoreceptor degeneration (Mancuso et al., 2009). Indeed, intraocular injections of adeno-associated virus vectors can be used to convert marmoset ganglion cells and other inner retinal cell types into photosensitive cells, by expression of channelrhodopsins (Ivanova et al., 2010). This may offer an approach for development of treatments for blindness caused by retinal degenerative diseases.

### OTHER RETINAL NEURONS AND OUTPUT PATHWAYS

Parallel pathways emerge in the output of cone photoreceptors, which in primates distribute their signals to at least nine different classes of bipolar cells (Boycott and Wässle, 1991; Chan et al., 2001). These in turn provide input to at least 15 morphological classes of retinal ganglion cell (Percival et al., 2009, 2011, 2013; Moritoh et al., 2013). In the marmoset, the peak ganglion cell density is ∼550,000 ganglion cells/mm^2^, so each foveal cone is sampled by at least two ganglion cells (Wilder et al., 1996). These parallel pathways within the retina, and their subsequent targets in the brain, are remarkably similar in macaques and marmosets. Criteria used for morphological classification of horizontal cells, bipolar cells, amacrine cells, and ganglion cells in macaques are generally just as suitable for classification of the same cells in marmosets (Ghosh et al., 1996; Chan et al., 1997, 2001; Chan and Grünert, 1998; Jusuf et al., 2004; Szmajda et al., 2008), providing that the smaller eye and retina of the marmoset are taken into account. Some differences in protein expression (assessed by antibody binding) are apparent, but these appear minor (Chan et al., 2001; Puller et al., 2014). Specifically, antibodies to recoverin stain flat midget bipolar cells in macaque but do not stain any bipolar cells in marmoset retina; antibodies to the carbohydrate epitope CD15 stain only DB6 cells in macaque retina but stain two populations of bipolar cells in marmoset (Andressen and Mai, 1997; Chan et al., 2001). It is not known if there are functional correlates of these differences in expression. Recent work has successfully developed organotypic tissue culture of the marmoset retina (Moritoh et al., 2013; Percival et al., 2014). This method gives a new complementary line of analyses of the retinal circuitry underlying parallel visual pathways.

As in all mammals studied to date, most ganglion cells in the marmoset retina can be classified as “ON-center” or “OFF-center” (Protti et al., 2014). A smaller number of ganglion cells respond well to both the onset and offset of light (“ON–OFF”). Retinal ganglion cells generally show classical center-surround receptive field organization, with a smaller excitatory center surrounded by a larger inhibitory surround. This center-surround organization is already present in the bipolar cells that provide excitatory input to ganglion cells, and the surround of ganglion cells is likely augmented by amacrine cells in the inner retina (Protti et al., 2014).

**Figure 2:**
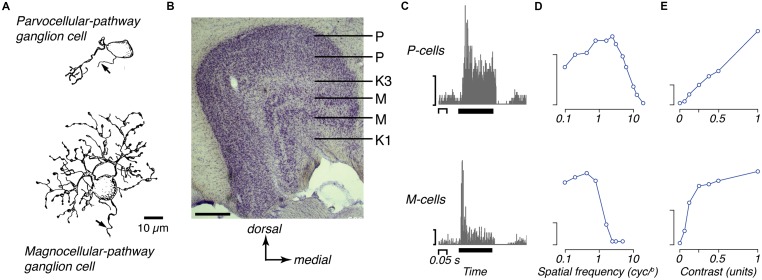
**The two major retino-thalamic pathways in marmoset. (A)** Camera lucida drawings of representative midget (parvocellular-pathway) and parasol (magnocellular-pathway) ganglion cells in marmoset retina, each located about 1 mm from the fovea (reproduced from Ghosh et al., 1996). **(B)** Photomicrograph of the LGN, showing the pairs of parvocellular (P) and magnocellular (M) layers; the dorsal most P layer and ventral most M layer get input from the contralateral eye; the internal layers get input from the ipsilateral eye. These layers are embedded in a matrix of koniocellular cells that lie between the principal layers, including two prominently segregated zones (K1, K3). Scale bar = 0.5 mm. **(C)** peristimulus time histograms of the responses of representative OFF P- and M-cells to brief (0.2 s) decrements in light from a gray background. The P-cell shows sustained response, the M-cell shows transient response (reproduced from Cheong and Pietersen, 2014). Y-axis scale bars 50 impulses/s. Thick black bar shows the time and duration of the stimulus. **(D)** Spatial-frequency tuning of representative P- and M-cells for drifting achromatic gratings, modulated at 4 Hz (adapted from White et al., 2001). Y-axis scale bars 20 impulses/s. **(E)** Contrast response of representative P- and M-cells for drifting gratings of optimal spatial frequency (adapted from Cheong and Pietersen, 2014).

Around 90% of the ganglion cells project to the lateral geniculate nucleus (LGN) of the thalamus (Jusuf et al., 2006b; Szmajda et al., 2008). The LGN of the marmoset has a basic laminar organization, which emerges before birth (Garey and de Courten, 1983). The size of the LGN increases rapidly after birth, without an increase in the number of neurons, and stabilizes at about 6 months of age (Fritschy and Garey, 1986b, 1988). Retinal input arrives mainly at two dorsal parvocellular layers and two ventral magnocellular layers, each receiving dominant input from either the contralateral or the ipsilateral eye. These layers are embedded in a matrix of smaller koniocellular neurons (**Figure 2**; Le Gros Clark, 1941; Kaas et al., 1978; Spatz, 1978; Solomon, 2002). In the marmoset koniocellular neurons are well segregated from the principal layers in two particular zones, one ventral to the magnocellular layers (K1), and one between the internal parvocellular and magnocellular layers (K3). This segregation has allowed targeting of koniocellular zones for electrophysiological recordings (see below) and anatomical tracing, so much of what we know about the koniocellular visual pathways in simian primates stems from work in marmoset.

Most retinal ganglion cells are of the midget class, and project to the parvocellular layers of the LGN (Goodchild et al., 1996; Gomes et al., 2005; Jusuf et al., 2006a). Within about 10° of the fovea, ON- and OFF-type midget ganglion cells appear to get input from a single midget bipolar cell (Ghosh et al., 1996; Goodchild et al., 1996; Telkes et al., 2008), which in turn receive input from a single cone photoreceptor (Chan et al., 2001). Thus the midget-parvocellular system provides a way in which the signals of individual cone photoreceptors located in and near the fovea can be passed largely independently to the LGN. Note, however, that while in macaque the midget bipolar cells contact single cones out to at least 8 mm (40°), in marmosets the midget bipolar cells get convergent input from multiple cones at eccentricities above 1 mm (8°; Wässle et al., 1994; Telkes et al., 2008). In addition the density of ganglion cells falls more rapidly with eccentricity in marmoset than macaque (Wilder et al., 1996).

The ON and OFF parasol ganglion cells form the next most populous class of ganglion cell; these draw on multiple diffuse bipolar cells (Chan et al., 2001; Gomes et al., 2005; Eriköz et al., 2008) and project to the magnocellular layers of the LGN (Szmajda et al., 2008). The number of bipolar cells, and thus cone photoreceptors, converging onto a single midget or parasol ganglion cell increases with distance from the fovea (Jusuf et al., 2006b; Telkes et al., 2008). Neurons in the parvocellular and magnocellular layers project to V1 (Solomon, 2002; Cheong and Pietersen, 2014) and there are about as many LGN neurons projecting to V1 as there are likely retinal afferents to the LGN (ca. 400,000; Fritschy and Garey, 1986b; Solomon, 2002), suggesting that there is limited mixing of retinal signals in the LGN. This is consistent with simultaneous recordings from nearby LGN cells, which show little evidence of common retinal input (Cheong et al., 2011).

One well established pathway through the koniocellular zones of the LGN is that formed by the small bistratified ganglion cell, which in the macaque retina is characterized by strong blue–yellow color sensitivity (Dacey and Lee, 1994). Anatomical work shows very similar retinal morphology and connectivity for a small bistratified ganglion cell type in the marmoset (Ghosh et al., 1996, 1997; Ghosh and Grünert, 1999), which projects to the koniocellular zones of the LGN, particularly K3 (Szmajda et al., 2008). As described below, recordings from the dorsal koniocellular zones in the marmoset LGN, particularly K3, show the presence of neurons with blue–yellow color sensitivity (Martin et al., 1997; White et al., 1998); these neurons can be antidromically activated by electrical stimulation of V1 (Cheong and Pietersen, 2014), to which many koniocellular LGN neurons project (Solomon, 2002). The characteristics of other retinal ganglion cells projecting to the koniocellular layers are less well defined, although for some, their retinal morphology and laminar projection is becoming clearer (Szmajda et al., 2008; Percival et al., 2013). Recent work suggests that the ventral koniocellular zone (K1) is a particular target of the narrow thorny ganglion cell class (Percival et al., 2014). Neurons in this region can project to extrastriate regions of the visual cortex (Warner et al., 2010), and this network potentially provides a direct route from the retina to extrastriate cortex, which mediates residual visual capabilities following lesions of V1 (Rodman et al., 1989; Rosa et al., 2000; Yu et al., 2013).

Anterograde labeling techniques show that there are substantial projections from the retina to non-geniculate thalamic areas including the pulvinar complex (Warner et al., 2010), pregeniculate nucleus (potentially homologous to the intrageniculate leaflet and ventral geniculate nucleus of rodents: Lima et al., 2012), and smaller projections to the midline and dorsomedial thalamic nuclei (Cavalcante et al., 2005; de Sousa et al., 2013). There are also projections from the retina to the accessory optic system, including the medial terminal nucleus (Weber and Giolli, 1986). Retinal projections to the hypothalamus include the suprachiasmatic nucleus, as well as diffuse projections to several other regions (Costa et al., 1999). Systematic studies of the retinal projection to the superior colliculus, nucleus of the optic tract, and pretectum, among others, are lacking. The organization of ganglion cells that comprise these non-geniculate pathways has also not been clarified in the marmoset. Intrinsically photosensitive retinal ganglion cells (which express melanopsin) are morphologically similar in marmosets and macaques (Jusuf et al., 2007). Their central projections include the LGN (Szmajda et al., 2008), but other targets are possible.

## FUNCTIONAL PROPERTIES OF NEURONS IN THE SUBCORTICAL VISUAL SYSTEM

There is now a substantial body of work describing the functional properties of neurons in the retino-geniculate pathway, as we review below. Among subcortical areas other than the LGN neuronal recordings have only been reported from superficial layers of the superior colliculus (Tailby et al., 2012; see Bourne and Rosa, 2003a for a description of the laminar organization of this nucleus). Parvocellular, magnocellular, and koniocellular neurons are generally well segregated in the marmoset LGN (Kaas et al., 1978; Bourne and Rosa, 2003b), allowing correlation of functional properties with the anatomical position of recorded neurons. In particular, work in the marmoset suggests that the functional properties of neurons in the parvocellular and magnocellular layers are each relatively homogenous, whereas neurons in the koniocellular zones form a more heterogeneous population.

Extracellular recordings from the LGN, generally obtained under opiate anesthesia, show that the receptive fields of neurons are very similar to those in macaques (**Figure 2**; Kremers et al., 1997, 2001; Kremers and Weiss, 1997; Solomon et al., 1999; White et al., 2001; Solomon et al., 2002; Forte et al., 2005). Neurons in the parvocellular layers have small receptive fields, low contrast sensitivity, a generally linear contrast–response function and a sustained response to an effective stimulus. Neurons in the magnocellular layers have larger receptive fields, higher contrast sensitivity, saturating contrast response function and a transient response to an effective high contrast stimulus. Magnocellular cells show contrast adaptation, such that sensitivity drops during prolonged presentation of an effective stimulus (Camp et al., 2009, 2011). Magnocellular neurons also show presence of a strongly suppressive region surrounding the classical receptive field (Felisberti and Derrington, 2001; Solomon et al., 2002; Webb et al., 2002, 2005; Kilavik et al., 2003; Kremers et al., 2004). Neurons in the parvocellular layers are less susceptible to contrast adaptation, and show weaker suppressive surrounds.

Measurements with drifting gratings reveal that the receptive fields of parvocellular neurons in the parafovea are usually less than 0.1° in diameter, and can resolve greater than 10 cycles/degree (Kremers and Weiss, 1997; White et al., 2001; Martin et al., 2011). Magnocellular neurons have larger receptive fields; because they are very sensitive to contrast their spatial resolution can be as high as that of parvocellular neurons for low contrast stimuli (White et al., 2001). Among both parvocellular and magnocellular neurons, receptive field size increases with distance from the fovea and the response becomes more transient; however, at any given eccentricity, magnocellular neurons have larger receptive fields, shorter visual latencies, and more transient responses than parvocellular neurons (White et al., 2001; Solomon et al., 2002; Pietersen et al., 2014; see also Silveira and de Mello, 1998).

The presence of dichromatic and trichromatic individuals makes the marmoset a natural model to study normal red–green color vision, anomalous color vision and color-blindness. Recordings from parvocellular neurons in the LGN show that if an individual female expresses two photoreceptor opsins in the middle-long wavelength range (see above) then cone-opponent receptive fields can be identified, as long as the receptive fields are close to the fovea (Yeh et al., 1995; White et al., 1998; Blessing et al., 2004; Martin et al., 2011). The chromatic properties of these receptive fields are very similar to those of parvocellular-pathway neurons in the macaque and there is no evidence that the presence of red–green color responses in trichromatic animals is associated with a change in the achromatic response properties of cells in the retino-geniculate pathway (Blessing et al., 2004; Victor et al., 2007; Martin et al., 2011). That achromatic signals are independent of chromatic signals in parvocellular cells is consistent with the idea that chromatic processing is achieved by mechanisms that are primarily concerned with spatial analysis (Ingling and Martinez-Uriegas, 1983; Paulus and Kröger-Paulus, 1983). Overall, however, the segregation of cone-opponent inputs to center and surround of the receptive field is more pronounced in macaque than in marmoset (Buzás et al., 2006). This may reflect higher convergence of cone photoreceptors onto the receptive fields of ganglion cells outside of the fovea.

Neurons in koniocellular zones of the marmoset LGN show diverse response properties. Many respond well to achromatic stimuli (Solomon et al., 1999), and their receptive fields are generally larger than those of parvocellular and magnocellular neurons at the same eccentricity from the fovea (White et al., 2001). Some are “ON–OFF” (White et al., 2001; Solomon et al., 2010), some are suppressed by the presence of any stimulus (Solomon et al., 2010), and some are selective for orientation (Cheong et al., 2013). The most prominent functional characteristic is that many koniocellular neurons in K3 and K4 show strong functional input from short wavelength (S-) cones, responding well to an increase (“blue-ON”; Martin et al., 1997; White et al., 1998; Hashemi-Nezhad et al., 2008; Tailby et al., 2008, 2010) or decrease (“blue-OFF”; Szmajda et al., 2006; Tailby et al., 2008; Solomon et al., 2010) in S-cone activation. A small subset of neurons in and around the magnocellular layers shows highly non-linear spatial summation (White et al., 2001), although it remains unclear if these are a subset of magnocellular neurons, or part of a koniocellular pathway. Finally, koniocellular cells in the LGN show slow rhythms in spiking activity (Cheong et al., 2011). Spiking activity of nearby koniocellular cells waxes and wanes at the same time, and these slow rhythms appear to be correlated with changes in the EEG state as measured in the visual cortex. The meaning of this slow rhythm is unknown, and it is not known if the phenomenon is common to marmosets and macaques.

## PRIMARY VISUAL CORTEX (V1)

### STRUCTURE AND TOPOGRAPHIC ORGANIZATION

**Figure 3:**
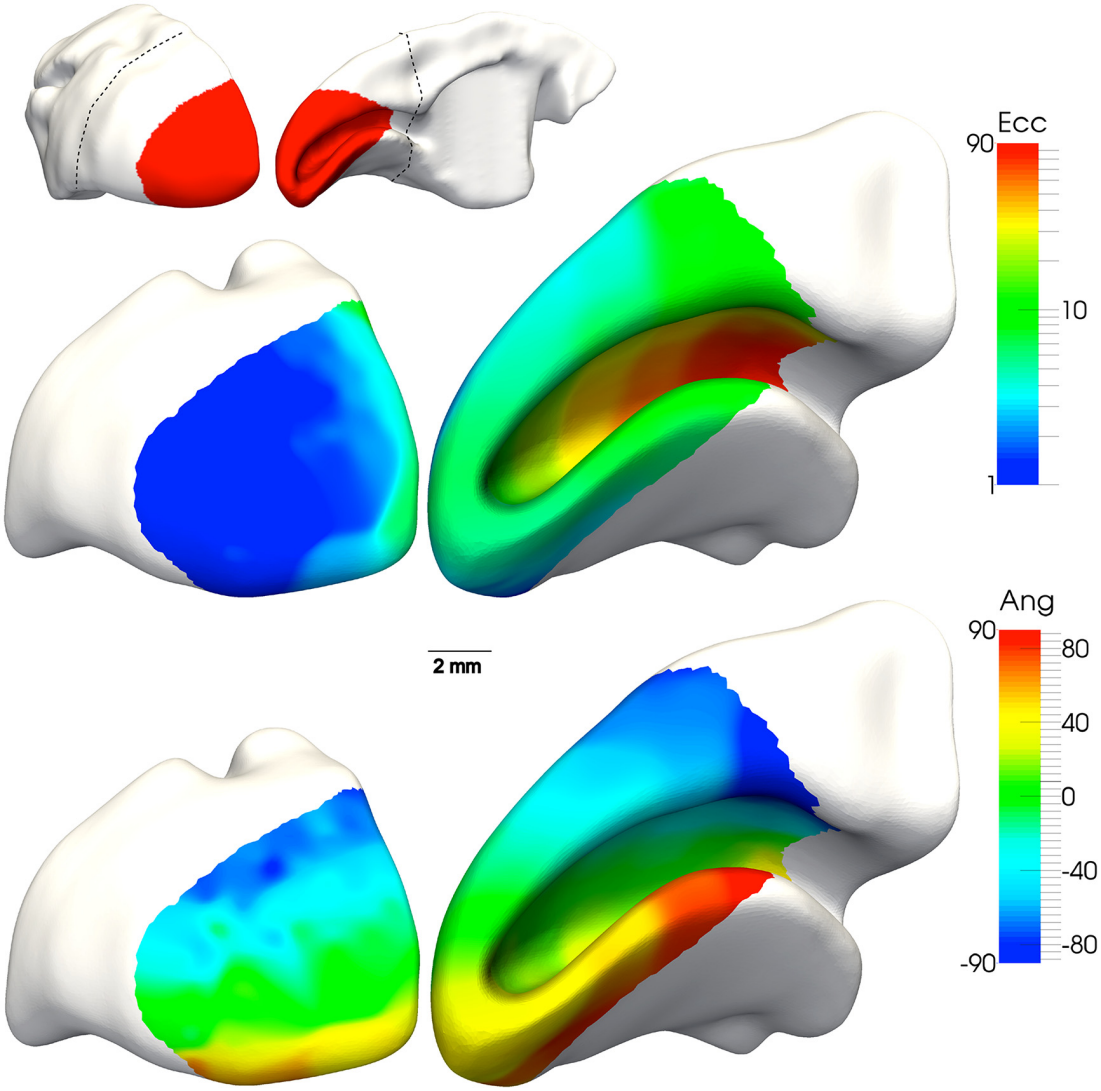
**Location and visuotopic organization of marmoset primary visual cortex (V1).** Top: caudal and medial views of the marmoset cerebral cortex, showing the location of V1 (red). The dashed line indicates the region reconstructed in the bottom panels. Middle**:** The representation of eccentricity from the fovea (“*Ecc*,” in degrees of visual angle), according to the color scale shown on the right. This reconstruction represents data from a single individual, in which hundreds of recording sites were obtained (Chaplin et al., 2013a). The portion of V1 exposed on the caudal surface of the brain corresponds to the representation of the fovea and parafovea (dark blue), while the far periphery of the visual field is represent at the most anterior portion of the calcarine sulcus (red). Bottom: The representation of polar angle (“*Ang*”) in the same individual. The lower contralateral visual field (blue, cyan) is found on the dorsal surface, and the upper contralateral field (yellow, orange, red) is found on the ventral surface. The representation of the horizontal meridian (green) divides V1 nearly equally.

V1 is the largest single area in the marmoset brain, with a surface area of approximately 200 mm^2^ in each hemisphere (Pessoa et al., 1992; Missler et al., 1993a; Fritsches and Rosa, 1996). Marmoset V1 is also very large in relative terms in comparison with that in other species of monkey, including the macaque (20% versus 10% of the total area of the neocortex; Rosa and Tweedale, 2005; Chaplin et al., 2013b). The retinotopic map found in V1 of the marmoset is very similar to that described for the macaque and other diurnal primates (Fritsches and Rosa, 1996; Schira et al., 2012; Chaplin et al., 2013a; **Figure 3**). The foveal representation is highly magnified, occupying ∼20% of the surface area, and about 60% of V1 is dedicated to the central 10° of the visual field (Chaplin et al., 2013a). The peak magnification factor near the representation of the center of the fovea has been estimated to be 4–5 mm/degree, about 40% of the equivalent value in the macaque (Van Essen et al., 1984; Dow et al., 1985), and this proportional relationship is maintained throughout the visual field. The representations of the upper and lower contralateral quadrants are nearly symmetrical in size. As in other primates (e.g., Silveira et al., 1989; Azzopardi and Cowey, 1993), the magnification factor follows the sampling density of ganglion cells, but detailed analysis show that representation of the foveal field in V1 greatly exceeds that expected based from the retinal ganglion cell density (Chaplin et al., 2013a). This magnification of central vision in V1 is likely due to greater divergence in the retino-geniculo-cortical pathways serving foveal vision, compared to those serving peripheral vision (Chaplin et al., 2013a).

**Figure 4:**
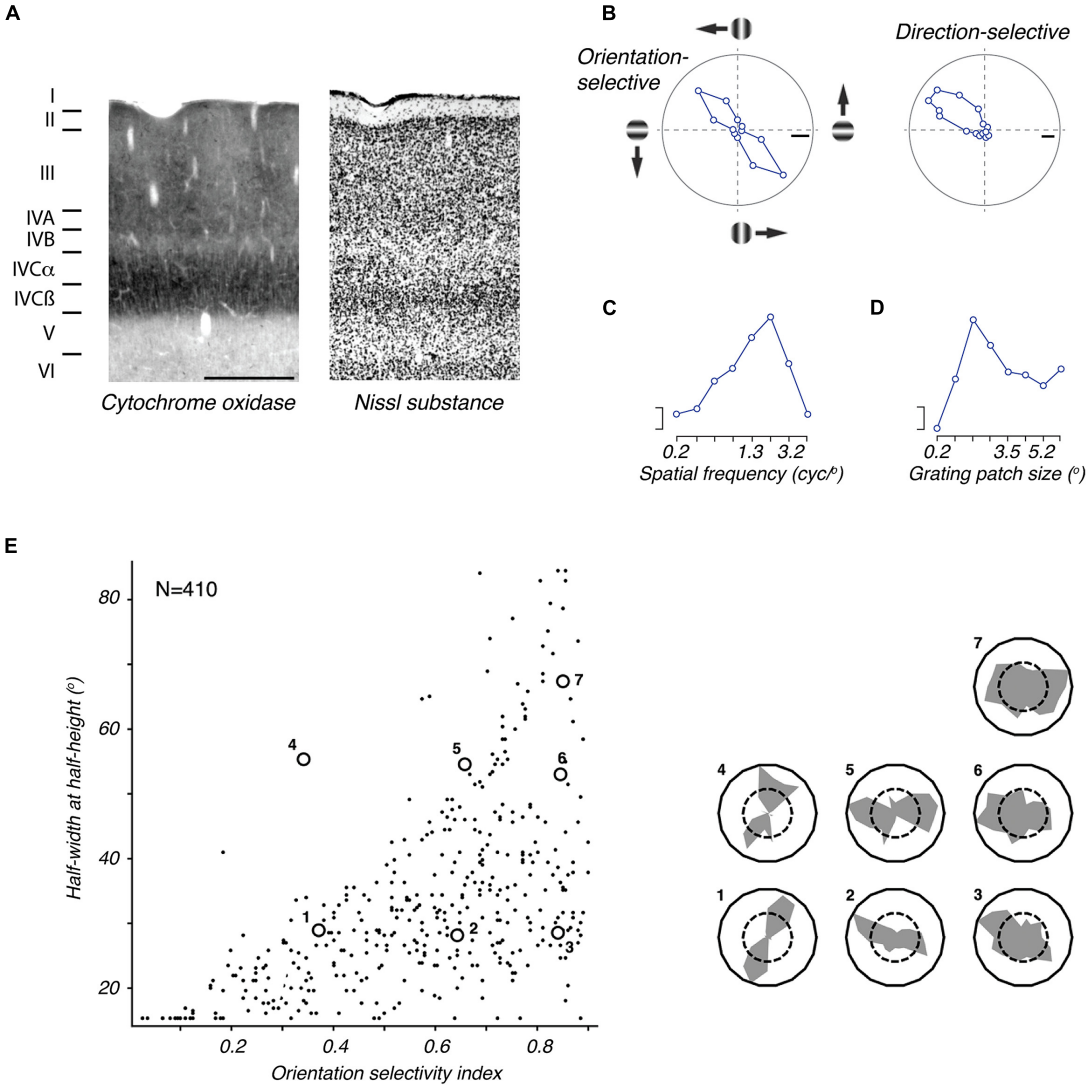
**The primary visual cortex (V1) of marmoset. (A)** Photomicrographs of neighboring coronal sections through V1, showing the laminar structure as revealed by staining for cytochrome oxidase (left) and Nissl substance (right). Scale bar = 0.5 mm. Reproduced from Solomon (2002). The terminology of layers follows that defined by Brodmann. **(B)** Tuning for grating orientation and direction in two representative V1 neurons. Left: orientation selective neuron, responding equally well to gratings of appropriate orientation, in both directions of drift (adapted from Cheong et al., 2013). Right: direction selective neuron (adapted from Tinsley et al., 2003). **(C)** Spatial frequency tuning of representative parafoveal V1 neuron (adapted from Yu and Rosa, 2014); the response to low spatial frequencies is negligible. **(D)** Tuning for the size of a patch of drifting grating, of optimal spatial frequency (adapted from Yu and Rosa, 2014): response is suppressed in large sizes, showing presence of extraclassical receptive field modulation, or suppressive surround. Scale bars in **(B–D)** show 20 impulses/s. **(E)** Distribution of orientation selectivity amongst V1 neurons in marmoset. The abscissa shows an orientation selectivity index based on the circular variance (higher numbers indicate poorer tuning); the ordinate shows half-width at halfheight of a von Mises function fit to the tuning curve. The inset at right shows orientation tuning of example neurons that are indicated in the plot. Adapted from Yu and Rosa (2014).

The laminar organization of marmoset V1 (**Figure 4A**) is similar to that seen in other diurnal primates, as revealed by the distribution of Nissl stain, and several neurochemical markers (Gebhard et al., 1993; Spatz et al., 1994; Goodchild and Martin, 1998; Solomon, 2002; Bourne et al., 2007). Although the layers of V1 are fully formed at birth, many important developmental events occur postnatally, with marked changes particularly within the first 3 months (Missler et al., 1993a,b; Spatz et al., 1994; Bourne et al., 2005; Fonta et al., 2005; Ribic et al., 2011). The reader should note that some studies (e.g., Spatz, 1975a; Vogt Weisenhorn et al., 1995; Elston et al., 1996, 1999; Solomon, 2002; Bourne and Rosa, 2003b) have employed a nomenclature of cortical layers in V1 that differs from the more commonly used Brodmann scheme (Hassler, 1966; see Casagrande and Kaas, 1994 for a discussion of the relative merits of the two schemes). The main difference to keep in mind is that in the Hassler scheme the layers IVa and IVb of the Brodmann nomenclature are considered subdivisions of layer III.

Relatively little is known about the distribution of cell types and interlaminar connections in marmoset V1. The few studies that have addressed neuronal morphology in this area have concentrated primarily on dendritic architecture, with respect to columnar domains (Malach, 1992), projection patterns (Vogt Weisenhorn et al., 1995; Elston and Rosa, 2006) or postnatal development (Fritschy and Garey, 1986a; Oga et al., 2013). One possible point of interest is the fact that most, if not all layer IVb cells, which form the projection to the middle temporal area (MT), have an unambiguously pyramidal morphology (Vogt Weisenhorn et al., 1995; Elston and Rosa, 2006), as opposed to spiny multipolar in the macaque (Yabuta et al., 2001; see, however, Elston and Rosa, 1997).

### CONNECTIONS OF V1

Perhaps surprisingly, our knowledge of the afferent connections of V1 in the marmoset still has many gaps. As expected from studies in other simian primates, anterograde tract tracing has shown strong projections from the LGN to layers IVcα (IVα in Hassler’s nomenclature) and IVcβ (IVβ), as well as a weaker projection to layer VI, and patchy projections to supragranular layers (Spatz, 1979; DeBruyn and Casagrande, 1981). Analysis of retrograde tracing shows that the projection to supragranular layers arises primarily from koniocellular LGN neurons, whereas parvocellular and magnocellular LGN neurons project primarily to layers IV and VI (Solomon, 2002). A projection from the lateral pulvinar complex to V1 has been demonstrated, but its laminar targets have not been determined (Dick et al., 1991). Other subcortical projections to marmoset V1 have not yet been investigated in any detail.

Substantially more research is also needed on the issue of the intrinsic connectivity of V1 in the marmoset. Knowledge of horizontal connections would specify how signals are pooled across visual space and functional domains (e.g., orientation columns): to date, we know only that periodic horizontal connections have been shown between neurons in supragranular layers (Solomon, 2002), which have similar periodicity to the distribution of cytochrome oxidase “blobs.” Knowledge of intralaminar connections would help specify the flow of information through V1 (e.g., Douglas and Martin, 1991), but the distribution of interlaminar connections has remained virtually unexplored, with the exception of a demonstration of projections from layer VI to the superficial layers (I and II; Divac et al., 1987).

Additional inputs to V1 arise in “feedback” connections from various other cortical areas. These connections originate primarily from infragranular layers in those areas (e.g., Spatz, 1977; Rosa and Tweedale, 2000), but their precise laminar targets in V1 have not been determined. Feedback projections originate mainly from other topographically organized areas, but also include smaller projections from subdivisions of the caudal parietal and inferior temporal cortices (Rosa and Tweedale, 2000; Lyon and Kaas, 2001). No study has mapped the entire pattern of extrastriate projections to V1, but injections into the central visual field representation (Rosa and Tweedale, 2000; Lyon and Kaas, 2001) label neuronal projections from V2, the ventrolateral posterior (VLP) and ventrolateral anterior (VLA) areas (likely homologs of areas V3 and V4 in the macaque; Rosa and Manger, 2005), the dorsomedial area, DM (V6; Rosa et al., 2013), MT (V5), and the middle temporal crescent [MTC; V4 transitional (V4t)]. Less dense, but clear projections were also detected from the dorsoanterior area, DA (a likely homolog of V3a; Rosa and Schmid, 1995) and other areas forming the occipitoparietal transition, as well as the caudal inferior temporal cortex (ITc). Overall, this pattern conforms to that described by studies using fluorescent tracers in the macaque (Perkel et al., 1986; Rockland and Van Hoesen, 1994; Markov et al., 2014).

Knowledge about the projection of V1 to extrastriate cortex in the marmoset comes mainly from retrograde tracer injections in extrastriate areas, which suggest that, as in other primates, V1 sends reciprocal projections to most, if not all areas from which it receives afferents (e.g., Spatz, 1977; Krubitzer and Kaas, 1990; Lyon and Kaas, 2001; Rosa et al., 2005, 2009; Palmer and Rosa, 2006a,b). Projections to V2 arise throughout the upper layers of V1 (from layer II to layer IVb), but there is also a small projection from layer VI. As in macaques, layer IVb (IIIc in the Hassler nomenclature) contains the majority of neurons that project to thick cytochrome oxidase “stripes” in area V2 (Federer et al., 2009, 2013). In addition, layer IVb is also the primary source of V1 input to areas MT (Spatz, 1977; Palmer and Rosa, 2006a) and DM (Rosa et al., 2009; Jeffs et al., 2013); however, the morphology of cells projecting to MT and DM differs in detail (Vogt Weisenhorn et al., 1995).

Finally, callosal fibers provide interhemispheric connections between left and right V1, which may be important in linking the representations of the left and right visual hemifields (Choudhury et al., 1965). These callosal connections appear to be more extensive than those reported in the macaque (Cusick et al., 1984; Spatz and Kunz, 1984; Rosa and Manger, 2005). Most callosal neurons are found along the border between V1 and V2 (the representation of the vertical meridian), but can also be found more than 1 mm within V1.

### COLUMNAR ORGANIZATION OF V1

The presence or absence of ocular dominance columns (ODCs) in marmosets remains a matter of interest. Early work suggested that marmosets lack ODCs in adulthood (Spatz, 1979, 1989; DeBruyn and Casagrande, 1981), although they can be transiently induced by silencing the input from one eye (Markstahler et al., 1998). Functional measurements in adults also suggest weak segregation of ocular dominance (Sengpiel et al., 1996; Roe et al., 2005). It is likely that ODCs form transiently during development (Spatz, 1989; Chappert-Piquemal et al., 2001): monocular lid suture during development can stabilize these ODCs into adulthood (Sengpiel et al., 1996). Transient or unstable expression of ODCs in marmosets is consistent with observations in some other New World monkeys, where the pattern and presence of ODCs varies from animal to animal (Adams and Horton, 2003). This variability may suggest that expression of ODCs is not necessary, or does not advantage any particular visual function; rather, the segregation of ocular inputs observed in adults of some primate species may simply reflect “leftovers” of a stochastic developmental process (Horton and Adams, 2005). Strong evidence for functional ODCs, in electrophysiological or optical imaging experiments, has not been reported in any individual marmoset (Sengpiel et al., 1996; Schiessl and McLoughlin, 2003; Roe et al., 2005).

As in cats and macaques, but unlike in rodents, V1 in the marmoset shows a columnar organization of orientation preference. Optical imaging reveals regions of relatively homogenous orientation preference (“iso-orientation domains”) interspersed with regions of rapid change (“pinwheels”; Liu and Pettigrew, 2003; Roe et al., 2005; McLoughlin and Schiessl, 2006; Buzás et al., 2008; Valverde Salzmann et al., 2011).

The upper layers of marmoset V1 are also characterized by patchy (“blob” like) distribution of staining for cytochrome oxidase, a marker of metabolic activity, and these blobs align with the axon terminals of koniocellular LGN neurons (Solomon, 2002; Roe et al., 2005; Federer et al., 2009; Valverde Salzmann et al., 2012). Neurons in blobs are often thought to be important for color vision, but there is no difference in the distribution of blobs in dichromatic and trichromatic marmosets (Solomon, 2002). Optical imaging studies of spatial organization of chromatic responses in the marmoset have found no spatial organization of the blue–yellow chromatic response or the achromatic response across the cortical surface (Roe et al., 2005; Buzás et al., 2008; Valverde Salzmann et al., 2012). However, spatial non-uniformity has been identified in trichromatic animals, such that the “red–green” chromatic response is more likely to be found in cytochrome-oxidase “blobs” (Valverde Salzmann et al., 2012). Finally, whereas in Old World macaques and New World capuchin monkeys blobs lie at the center of ODCs (Livingstone and Hubel, 1984; Rosa et al., 1991), in marmosets, where such columns seem largely absent, blobs appear to form a hexagonal array (Solomon, 2002).

### FUNCTIONAL PROPERTIES OF V1 NEURONS

Although the literature on single unit response properties in the marmoset visual cortex is still small relative to that in the macaque, there has been substantial progress, particularly over the last decade. To date, analyses of the response properties in V1 of the marmoset have been made under either barbiturate (Sengpiel et al., 1996; Roe et al., 2005; McLoughlin and Schiessl, 2006) or, more commonly, opiate anesthesia. Quantitative measurements from visual neurons in awake marmosets are not yet available, but the recent demonstration of the animals’ ability to maintain fixation and perform visual tasks under head fixation (Mitchell et al., 2014), combined with the success of marmosets for single-unit recordings in other sensory systems (Lu et al., 2001; see Wang et al., 2008 for review), suggests that this situation will change substantially in the coming years. As we show below, there is little to differentiate the functional properties of neurons in V1 of marmosets and other primates.

The spatial response properties of marmoset V1 neurons strongly resemble those described in the macaque (**Figures 4B–D**). The degree of orientation selectivity varies between neurons (**Figure 4E**), but throughout V1 the majority of neurons (∼80%) show clear orientation preference. Quantitative analyses show that the orientation bandwidth (half width at half height) is on average 22–29° (Sengpiel et al., 1996; Bourne et al., 2002; Forte et al., 2005; Zinke et al., 2006; Cheong et al., 2013; Yu and Rosa, 2014). Some neurons in marmoset V1 show “simple” responses to drifting gratings, with the response modulated at the temporal frequency of the drift, and consistent with spatially offset ON and OFF subregions. The remainder shows “complex” responses to drifting gratings, with an increase in the mean rate but no modulation of discharge. In some studies the prevalence of simple cells is 5–15% (Sengpiel et al., 1996; Yu and Rosa, 2014); other work finds approximately equal prevalence of simple and complex cells (Webb et al., 2003; Forte et al., 2005; Nowak and Barone, 2009). The reason for this discrepancy is not clear, and may be related to specific conditions of the tests conducted (Crowder et al., 2007); the latter estimates are nearer those found in macaques.

The preferred spatial frequency (**Figure 4C**) among V1 neurons depends strongly on eccentricity from the fovea: preferred spatial frequency is ca. 1.1 cycles/degree within 5° of the fovea, and 0.14 cycles/degree at eccentricities beyond 50° (Sengpiel et al., 1996; Forte et al., 2005; Yu et al., 2010). At least for receptive fields in parafoveal visual space, the peak spatial frequency of V1 cells is comparable to that of marmoset LGN cells (Forte et al., 2005) and is about half that in macaque V1 (Foster et al., 1985), as expected from the smaller eye of the marmoset. Neurons in V1 are less responsive to low spatial frequencies and uniform fields than neurons in the LGN, and show correspondingly tighter bandwidth for spatial frequency (Forte et al., 2005; Martin et al., 2011).

Qualitative and quantitative analyses reveal that direction selectivity (**Figure 4B**), in response to either moving bars or drifting gratings, is evident in approximately 20% of marmoset V1 neurons (Sengpiel et al., 1996; Bourne et al., 2002; Yu and Rosa, 2014). These neurons are more likely to be found in the infragranular layers than the supragranular layers, and are absent from the granular layers (IVcα and IVcβ; Yu and Rosa, 2014). Most neurons are generally sensitive to motion orthogonal to the preferred orientation, and are incapable of extracting motion direction independent of contour orientation (Tinsley et al., 2003); the signals of some broadly tuned neurons are less dependent on contour orientation, and may be an early stage in complex motion analysis (Tinsley et al., 2003; see also Barraclough et al., 2006; Guo et al., 2006). On average, neurons in marmoset V1 prefer temporal frequencies of ca. 4 Hz throughout the visual field. In the central visual field, the preferred temporal frequency is generally independent of the spatial frequency, suggesting that the receptive fields of most neurons are not extracting a measure of retinal image speed. This may be different from the case in the macaque, where speed sensitivity in the corresponding region of V1 is apparent in a subpopulation of complex cells (Priebe et al., 2006). The proportion of neurons showing speed sensitivity increases in the peripheral visual field representation of marmoset V1 (Yu et al., 2010).

Neurons in marmoset V1 show a broad distribution of contrast sensitivity – some are sensitive to very low contrasts and others only respond at high contrast. Many neurons in marmoset V1 display a saturating contrast response function (Webb et al., 2003), which is usually taken as evidence for some form of contrast gain control. As in the macaque, other evidence for gain control is found in around half of V1 neurons, which show the presence of suppressive surrounds similar to those found in the LGN (**Figure 4D**). On average, making the stimulus larger than the preferred size reduces the response by about 30% (Webb et al., 2003; Bourne et al., 2004; Yu and Rosa, 2014). The large size of these suppressive surrounds makes many neurons selective for the size of a textured stimulus – the preferred size depends on eccentricity from the fovea, with a diameter of 1.4° in the parafovea, and about 10° at eccentricities beyond 50° (Webb et al., 2003; Yu and Rosa, 2014). Unlike in the LGN these surrounds can be orientation tuned: they are most evident during the presentation of gratings or contours that are aligned to the preferred orientation of the classical receptive field (Webb et al., 2003).

The majority of neurons in marmoset V1 can be driven by stimulation of either eye (Sengpiel et al., 1996), including those in layer IV. The percentage of binocular cells appears higher than that in macaques and other species of New World primate that show well-defined ODCs (Rosa et al., 1992). No study has yet investigated the sensitivity of neurons in the marmoset visual cortex to binocular disparity. The interocular distance of the marmoset is much smaller than that of larger primates; the range of depths that can be usefully discriminated from binocular disparity should be correspondingly smaller, but no behavioral or physiological evidence is currently available. Knowledge of disparity sensitivity early in the visual pathway will be necessary to understand mechanisms of depth perception in the marmoset.

There has been limited investigation of the chromatic response of neurons in marmoset V1. No study has characterized the response of V1 neurons to modulation along the red–green dimension of color space, which is present only in trichromatic animals; some work has investigated the response to blue–yellow modulation (Buzás et al., 2008; Hashemi-Nezhad et al., 2008). As in macaques, many neurons respond weakly to blue–yellow color but strong responses to blue–yellow color (that is, sensitivity similar to that of blue–yellow color-responsive cells in the LGN) are rare.

Finally, some of the spiking variability of cortical neurons is shared with other cortical neurons, as evidenced by correlations in the activity (“noise correlations”) of pairs of neurons. In V1 of marmoset, as in macaque, these noise correlations are dominated by short time-scales (<1 s), are slightly higher in pairs of neurons with similar functional characteristics, and extend over long distances (>1 mm; Cheong et al., 2011; Solomon et al., 2014).

## SECOND VISUAL AREA, V2

### STRUCTURE AND TOPOGRAPHIC ORGANIZATION

In common with other simian primates, marmoset area V2 forms a continuous belt that wraps around V1, except at the rostral end of the calcarine sulcus, where area prostriata is located (Rosa et al., 1997; **Figure 1**). The vertical meridian of the visual field is represented along the border with V1; the horizontal meridian is represented along the anterior border, where V2 abuts areas of the “third visual complex” (Jeffs et al., 2009; Rosa et al., 2013). Following the topology of V1, the lower visual field is represented in dorsal V2, and the upper visual field is represented in ventral V2. Whereas in the macaque V2 is nearly as large as V1 (Olavarria and Van Essen, 1997), in the marmoset it is only half as large, with a surface area of about 100 mm^2^ in each hemisphere (Rosa, 2002). The representation of the central visual field appears emphasized in V2, relative to V1, with approximately half of the surface area of V2 dedicated to the representation of the central 5° (Rosa et al., 1997).

### CONNECTIONS OF V2

There have been no detailed studies of the pattern of subcortical projections to marmoset V2, although early work confirmed that, as in most primates, thalamic afferents largely originate in the inferior and lateral subdivisions of the pulvinar complex (Dick et al., 1991), and are topographically organized (Kaske et al., 1991). In addition to the V1 input described above, major cortical afferents to V2 originate in the third visual complex (DM/V6 and VLP/V3), the fourth visual area (VLA/V4), the motion-sensitive areas MT and MTC, and other dorsal extrastriate areas (in particular, the dorsoanterior area, DA/V3a; Jeffs et al., 2009, 2013). These inputs are topographically organized. Much smaller projections to V2 arise from areas in the occipitoparietal transition (Jeffs et al., 2013), likely extending into the lateral intraparietal area (LIP), the fundus of the superior temporal area (FST), the caudal ITc (ITc/TEO), and the prefrontal cortex (primarily, area 8aV, which likely includes the frontal eye field; Burman et al., 2006; Reser et al., 2013).

### COLUMNAR ORGANIZATION OF V2

Like other simian primates (e.g., Livingstone and Hubel, 1984), marmoset V2 displays well-defined, stripe-like modular compartments, which are best visualized by stains for cytochrome oxidase (Rosa et al., 1997; Lyon and Kaas, 2001; Roe et al., 2005; Jeffs et al., 2009). Cytochrome oxidase-rich stripes can be further classified as thin or thick, which alternate with cytochrome oxidase-poor (or “pale”) interstripes. Each point in the visual field is sampled by a thin stripe, a thick stripe, and a pair of interstripes (Rosa et al., 1997). These stripes can also be defined by their inputs from V1. Neurons within V1 “blobs” project to thin stripes in V2, those at the borders of blobs project to the thick stripes, and those in the center of “interblob” regions project to interstripes. This last projection can be further distinguished, based on the laminar location of the V1 afferents, into parallel streams that target alternating interstripes (Federer et al., 2009). Specifically, “pale-lateral” interstripes receive 10% of their V1 input from layer IVb, while the “pale-medial” interstripes receive no IVb input; this finding that has recently been confirmed in macaque (Federer et al., 2013). Some of the details of connectivity between V1 and V2 may differ between marmosets and macaques, but the functional organization of this system in macaques remains a topic of ongoing debate (e.g., Livingstone and Hubel, 1984; Xiao and Felleman, 2004; Sincich et al., 2010; Federer et al., 2013).

### FUNCTIONAL PROPERTIES OF V2 NEURONS

Functional work on marmoset area V2 has been limited. The receptive field diameter of V2 neurons is 2–3 times greater than that in V1 (Rosa et al., 1997), but the neurons show a similar range of spatial and temporal properties, including orientation and direction selectivity, to those in V1 (Lui et al., 2005; Barraclough et al., 2006). The relationship with cytochrome oxidase modules has not been studied in detail, although one optical imaging study shows that regions with poor selectivity for orientation are coincident with “thin” cytochrome oxidase stripes, whereas regions with strong orientation selectivity coincide with the interstripes (Roe et al., 2005) and thick stripes (Federer et al., 2009). As in other primates (Malach et al., 1994), the imaged orientation domains in marmoset V2 are considerably larger than those in V1 (Liu and Pettigrew, 2003; McLoughlin and Schiessl, 2006).

## AREAS PROSTRIATA AND 23V

Area prostriata is a narrow (1–2 mm wide) belt of cortex that separates the representation of the far peripheral visual field in V1 from the hippocampal formation, near the rostral tip of the calcarine sulcus. Area prostriata is distinct from V2, with low myelination and a poorly developed layer IV. Similar to the macaque, in marmosets prostriata provides input to the peripheral representations of several visual areas, as well as to many other sensory and association areas, extending as far as the frontal pole (Palmer and Rosa, 2006b; Burman et al., 2011; Reser et al., 2013; see Yu et al., 2012 for review). Area prostriata is adjoined by area 23V (23 ventral), a subdivision of the posterior cingulate cortex with which it shares many connections, including projections to the peripheral representations of MT and the medial superior temporal area (MST; Palmer and Rosa, 2006b) and frontal visual association areas (Reser et al., 2013). Based on its location relative to V2, area 23V seems to correspond the scene-selective area of the retrosplenial cortex, described by Nasr et al. (2011) in other species.

Traditionally regarded as a high-order “limbic” visual association area, recent work in marmoset (Yu et al., 2012) suggests that area prostriata may be part of a primordial visual pathway parallel to that coursing through V1, which enables rapid response to events in peripheral vision and multisensory integration (Smiley and Falchier, 2009; Rockland, 2012). The subcortical afferents to this region are unclear, but neurons in area prostriata show short latency responses and broad tuning along the dimensions of orientation, direction, and spatial and temporal frequency; that is, their functional properties resemble those of neurons at early stages of visual processing. The receptive fields are, however, enormous (30–50° in diameter), and are concentrated in the peripheral visual field (Yu et al., 2012).

## “THIRD TIER” VISUAL CORTEX (AREAS DM, VLP, AND 19M)

**Figure 5:**
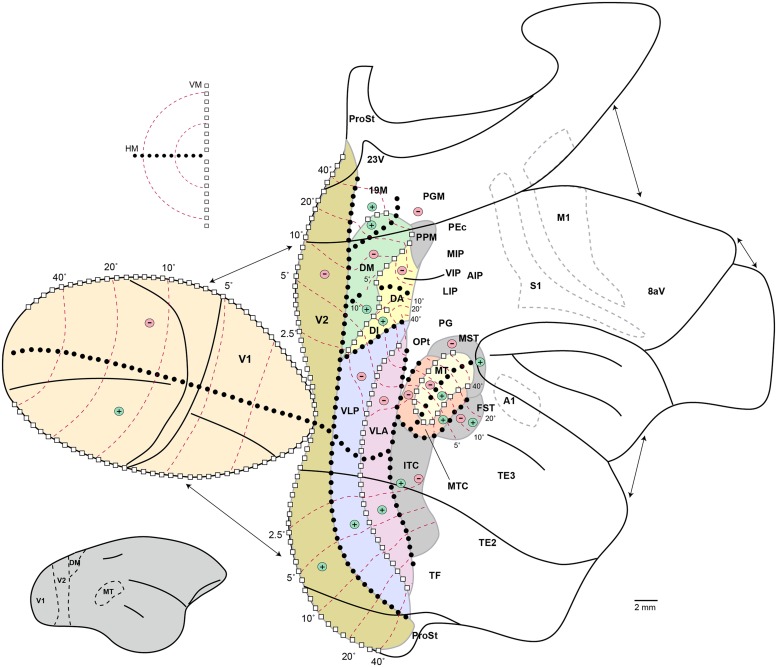
**Schematic organization of visual cortex in the marmoset.** “Unfolded” representation prepared using the technique of Van Essen and Maunsell (1980). Discontinuities in the representation, introduced to minimize distortion, are indicated by the arrows. Continuous black lines indicate the main cortical folds, including the lips and fundi of the lateral and calcarine sulci, the fundi of the superior temporal and intraparietal dimples, and the limits of the medial, ventral, and orbital surfaces. The inset on the lower left shows a lateral view of the intact marmoset brain, with boundaries of some visual areas indicated to help orientation. Colors indicate visual areas that have been mapped using electrophysiological techniques; other areas are simply indicated by labels in their approximate location. For abbreviations, see legend of **Figure 1**. The light gray dashed outlines indicate the borders of the primary auditory (A1), motor (M1), and somatosensory (S1) areas, for orientation. The topographic organization of visual areas is indicated according to the following symbols: white squares, representations of the vertical meridian (VM); black circles, representations of the horizontal meridian (HM); “+,” representations of upper contralateral quadrant; “-,” representations of the lower contralateral quadrant; red dashed lines, isoeccentricity lines (numbers indicate eccentricity from the fovea, in degrees).

The third tier visual areas are those that lie adjacent to the anterior border of V2, and in the marmoset these are exposed on the surface of the brain, rendering them more readily accessible to modern experimental techniques including multielectrode array recording, optogenetics, and imaging. Electrophysiological studies demonstrate at least two areas, each forming a near complete representation of the contralateral hemifield: areas DM (V6) and VLP (V3; **Figure 5**). Fragmentary evidence suggests the existence of at least one additional area, near the midline (19M; **Figure 1**). DM and VLP may also be separated by an anatomically distinct subdivision, the dorsointermediate area (DI; Krubitzer and Kaas, 1990; Rosa and Schmid, 1995; see **Figure 1**), about which virtually nothing is known.

### AREA DM

Area DM contains representations of the upper and lower visual fields, both of which lie adjacent to V2 (Rosa et al., 2005, 2013; Jeffs et al., 2013). At first sight, this organization seems to differ from that described in the corresponding region in the macaque brain, in which the dorsal cortex that is anterior to V2 is usually thought to contain only the lower visual field representation of area V3 (Gattass et al., 1988). However, anatomical evidence reveals strong similarities between marmoset DM and macaque area V6 [Rosa and Tweedale, 2001; Rosa et al., 2013; note that V6 overlaps partially with the “parietooccipital area” (PO) of other nomenclatures; Neuenschwander et al., 1994; Galletti et al., 2005]. Like macaque V6 marmoset DM is heavily myelinated, a characteristic which allows it to be easily distinguished from V2 and other subdivisions of the third tier complex, and obtains its predominant input from layer IVb neurons in V1; smaller projections arise in more superficial layers of V1 (Krubitzer and Kaas, 1993; Vogt Weisenhorn et al., 1995; Rosa et al., 2009; Jeffs et al., 2013). In addition, both marmoset DM and macaque V6 show a relatively large representation of the peripheral visual field, in comparison with most other visual areas.

In addition to the V1 projections, most cortical afferents to marmoset DM originate in extrastriate areas, including VLP and VLA, motion-sensitive areas MT, MTC, and MST, occipitoparietal transition areas [DA and PPM (medial posterior parietal area); see below], and other dorsal areas of the caudal posterior parietal cortex (in particular, LIP). Smaller cortical projections from the granular frontal cortex (primarily 8aV), rostral premotor cortex, ventral parietal cortex (primarily cytoarchitectural fields OPt and PG) and parahippocampal cortex (primarily TF) have also been described (Rosa et al., 2009; Jeffs et al., 2013; Burman et al., 2014b). Finally, subcortical projections from the pulvinar complex, centrolateral and centromedial thalamic nuclei, and claustrum, have been documented (Dick et al., 1991;Rosa et al., 2009).

The receptive fields of neurons in DM are about twice the diameter of those in V2 (Rosa and Schmid, 1995), although many neurons show larger, facilitatory, fields, suggesting a role in integrating contours across large regions of the visual field (Lui et al., 2006, 2013). Most neurons are orientation selective, and include some with remarkably narrow orientation tuning (Lui et al., 2006). Direction selectivity is observed in a minority of the neurons (Rosa and Schmid, 1995; Lui et al., 2006), although this deserves more careful study, particularly with respect to the peripheral visual field representation. These properties contrast sharply with those observed in MT, another densely myelinated area that receives projections from layer IVb of V1 (Lui et al., 2013).

### AREA VLP

Area VLP, which lies lateral to DM, is the likely homolog of the third visual area (V3, or area 19) found in most mammals (Rosa and Manger, 2005). In VLP the lower visual field is represented on the dorsolateral cortical surface, and the upper visual field on the tentorial surface (Rosa and Tweedale, 2000; Jeffs et al., 2013). Over half of VLP is devoted to the central 5° of the visual field, and there is little if any representation beyond 50°. The myeloarchitecture of VLP is similar to that of “ventral V3” (also known as the ventral posterior area, VP) in macaque and capuchin monkeys (Gattass et al., 1988; Rosa et al., 1993). Also similar to V3, the anterior border of VLP is formed by a representation of the vertical meridian of the visual field. VLP sends and receives topographically organized projections from the central visual field representations of areas V1 (Rosa and Tweedale, 2000; Lyon and Kaas, 2001), V2 (Jeffs et al., 2009, 2013), MT (Palmer and Rosa, 2006a,b), and DM (Rosa et al., 2009), but the full pattern of connections is yet to be determined. Quantitative measurements of response properties are not yet available, but direction selectivity is rare. Most cells prefer slow moving stimuli, and receptive fields are not much larger than those in area V2 (ca. 1° in diameter near the center of the fovea; Rosa and Tweedale, 2000). Preliminary evidence based on functional MRI suggests that VLP is closely affiliated with the ventral stream of visual processing (Ciuchta et al., 2013).

### Area 19M

Adjacent to the representation of the lower visual quadrant periphery of V2 (Rosa and Schmid, 1995), along the midline of the cortex, is area 19M (also named the “parietooccipital medial area,” POm). Area 19M lacks the heavy myelination that characterizes the adjacent DM, but shares with this area connections with MT and the frontal oculomotor fields (Palmer and Rosa, 2006b; Reser et al., 2013). The visual field representation encompasses the upper and lower visual fields, and the representation of the peripheral visual field seems expanded relative to that of V1 and V2. Area 19M is likely to overlap in part with the “medial visual area” described in the owl monkey (Allman and Kaas, 1976).

## MIDDLE TEMPORAL AREA, MT

### STRUCTURE AND TOPOGRAPHIC ORGANIZATION

**Figure 6:**
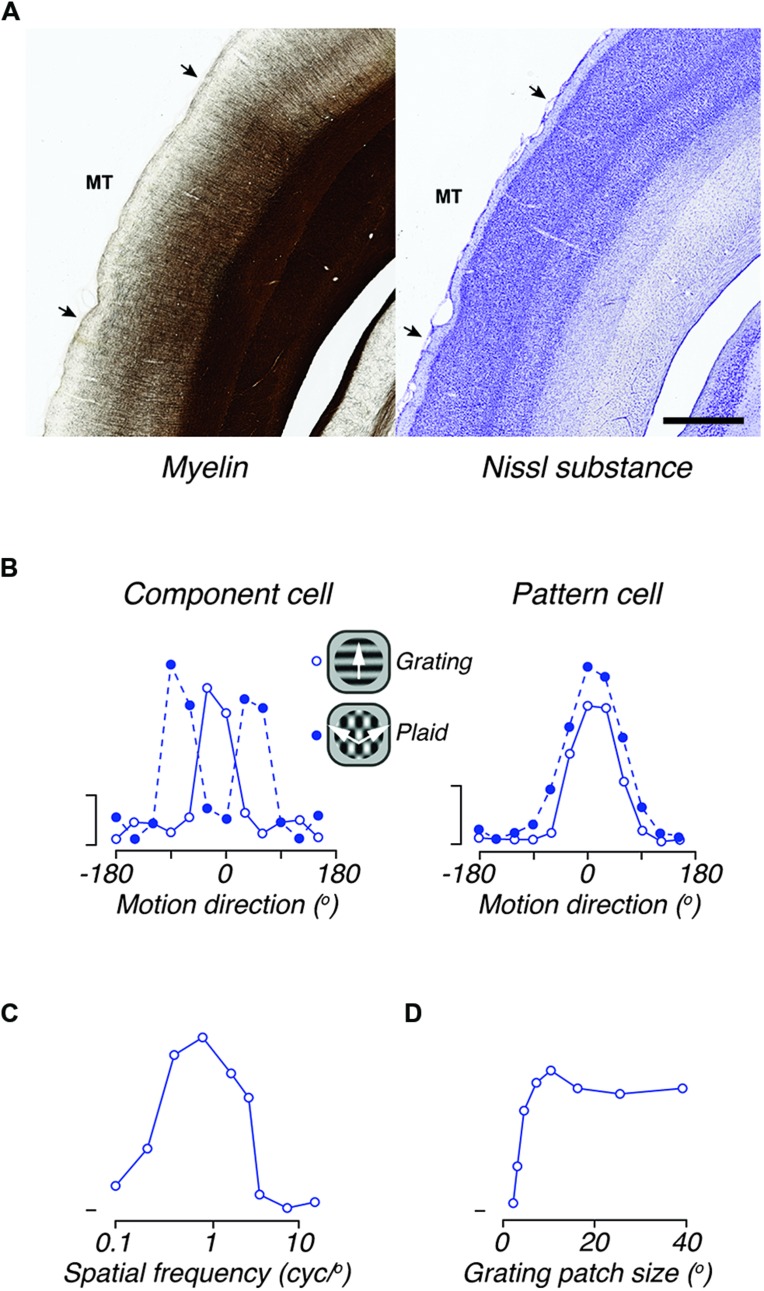
**The middle temporal area (MT) of marmoset. (A)** Photomicrograph of adjacent coronal sections, showing the histological distinctiveness of area MT revealed by myelin (left) and Nissl (right) stains. MT stands out as heavily myelinated in comparison with most cortical areas. Although the boundaries are less obvious, MT can also be identified in Nissl stained sections by the thinner and denser layer IV, and by the thicker layer VI, in comparison with adjacent areas. Scale bar = 1 mm. **(B)** Direction tuning for gratings and plaids in two representative directions elective MT neurons. The left panel illustrates the responses of a “component-cell,” which shows bi-lobed tuning for plaids, as if it responded to the individual gratings that comprise the plaid. The right panel shows the responses of a “pattern-cell,” which has similar direction tuning to gratings and plaids. **(C)** Spatial frequency tuning of a representative “component cell” in the peripheral representation of MT; the response to low spatial frequencies is neglible. **(D)** Tuning for the size of a patch of drifting grating, of optimal spatial frequency, showing large receptive field size of neurons in area MT. Scale bars in **B** show 20 impulses/s. **(B–D)** adapted from Solomon et al. (2011).

Area MT, which as in other primates is characterized by dense myelination (Spatz, 1977; Rosa and Elston, 1998; Bourne et al., 2007; Bock et al., 2009), lies posterior to the lateral sulcus (**Figures 1** and **Figures 6**). Marmosets (and probably other species of Callitrichidae) are the only simian primates in which MT is entirely exposed on the surface of the cortex, creating unique opportunities for studies using imaging, intracellular or multielectrode array analyses. The size of MT in the marmoset is approximately 13 mm^2^ in each hemisphere, making it about 6.5% the size of V1; these estimates are similar to those in other simian primates (Pessoa et al., 1992; Rosa, 2002). The representation of the central visual field is less emphasized than in V1: whereas the central 5° around the fixation point project to about 40% of the volume of V1, the corresponding region only occupies 20% of MT (Rosa and Elston, 1998).

### CONNECTIONS OF MT

The main thalamic afferents to MT originate in the inferior subdivision of the pulvinar complex, with smaller inputs from koniocellular layers K1 and K3 in the LGN (Dick et al., 1991; Warner et al., 2010, 2012), and intralaminar nuclei (Spatz, 1975b). Sparse projections also arise from the claustrum (Spatz, 1975b).

In addition to the V1 input described above, which primarily projects to lower layer III and upper layer IV of area MT (Spatz, 1977), major cortical afferents to MT originate in V2, in surrounding motion-sensitive areas (MTC, MST, and the fundus of superior temporal sulcus area, FST; Krubitzer and Kaas, 1990), and in other dorsal extrastriate cortex areas (in particular, DM, DA, 19M, and PPM). In comparison, input from ventral stream areas is minor (Palmer and Rosa, 2006a). Additional inputs arise in the posterior parietal cortex (primarily LIP), prefrontal cortex (primarily area 8aV; Reser et al., 2013), and parahippocampal cortex (TF). For quantitative analysis of these and other cortical projections, the reader is directed to Palmer and Rosa (2006a,b). Projections from area MT include a strong projection onto V1 (Spatz, 1977) and most, if not all areas from which it receives afferents (Krubitzer and Kaas, 1990). The pattern of intrinsic connections within marmoset area MT has not yet been explored.

### FUNCTIONAL PROPERTIES OF MT NEURONS

As in all primates so far studied, the connections and functional properties of area MT in marmoset are consistent with a role in motion analysis and the control of eye movements. The response properties of marmoset MT neurons strongly resemble those described in the macaque. The degree of direction selectivity varies between neurons, but throughout MT the majority of neurons (80–90%) show clear direction selectivity (**Figure 6B**), whether the stimulus is a moving grating, bar or dot field (Rosa and Elston, 1998; Solomon et al., 2011; Lui et al., 2013). Among these neurons there is a bias for motion radial from the fovea, particularly in the representation of the peripheral visual field (Rosa and Elston, 1998). Quantitative analyses show tuning bandwidth (half width at half height) of directionally selective neurons is around 33° for drifting gratings (Solomon et al., 2011) and slightly broader for moving bars, kinetic contours or dot fields (Solomon et al., 2011; Lui et al., 2012, 2013).

Direction-selective neurons in area MT of the macaque are distinguished from those in V1 by their capacity to signal motion direction independently of contour orientation. This is most commonly revealed by comparing responses to drifting gratings, and plaids formed by the superposition of two such gratings (**Figure 6B**). Some neurons respond to plaids with bimodal direction tuning curves, as if they “see” each of the components of the plaid (“component cells”), and others respond to the overall motion direction of the plaid and not that of its components (“pattern cells”); other neurons respond in an intermediate way. In both qualitative and quantitative aspects the signatures of this motion integration are the same in MT of marmosets and macaques (Solomon et al., 2011; McDonald et al., 2014).

Receptive field sizes in area MT are much larger than those in V1 (**Figure 6D**), and as in the macaque, the average receptive field diameter is similar to the receptive field eccentricity (i.e., a receptive field centered at 10° eccentricity will be about 10° wide). Each point in the visual field projects onto 1–1.5 mm of the surface of area MT (Rosa and Elston, 1998). Most neurons in marmoset MT show a “complex” response to drifting gratings, with an unmodulated increase in the mean firing rate (e.g., Solomon et al., 2011). The preferred spatial frequency (**Figure 6C**) depends weakly on eccentricity from the fovea: it is about 0.2 cycles/degree within 5° of the fovea, and 0.1 cycles/degree at eccentricities beyond 30° (Lui et al., 2007a). Neurons are generally insensitive to modulation of uniform fields, but show broad bandwidth for spatial frequency (Lui et al., 2007a; Solomon et al., 2011). The preferred temporal frequency is in the range 4–12 Hz, increasing in the peripheral field. In about one-third of neurons, the preferred temporal frequency depends on the spatial frequency, suggesting that the receptive fields of these neurons are extracting a measure of retinal image speed (Lui et al., 2007a). Responses to drifting dot-fields show that the speed tuning of neurons can appear low-pass, band-pass, or high-pass (Solomon et al., 2011).

Neurons in marmoset MT show very high contrast sensitivity, and a saturating contrast–response function, with the contrast to achieve a half-maximum response ca. 0.13 (Solomon et al., 2011). Many neurons also show the presence of “suppressive surrounds.” On average, making a grating patch larger than the preferred size (generally similar to receptive field size) reduces the response by 40–50% (Lui et al., 2007b; Solomon et al., 2011). The inhibitory surrounds of marmoset MT neurons are primarily aligned with the receptive field length (i.e., perpendicular to the optimal direction of motion), so that end-inhibition tends to be stronger than side-inhibition (Lui et al., 2007b, 2013).

Like many other visual cortical areas, MT in the marmoset lies exposed on the cortical surface and is accessible to multielectrode arrays. Recent work has exploited this anatomical convenience to measure the spatiotemporal distribution of neural correlations in anesthetized animals, and its impact on the neural codes that populations of neurons in MT can provide (McDonald et al., 2014; Solomon et al., 2014). This work shows that the spiking activity of neurons within about 1.5 mm of each other (that is, neurons with overlapping receptive fields) can be tightly synchronized (<0.05 s), and is stronger in neurons with similar direction preference (Solomon et al., 2014). Superimposed on this are slower correlations (with time scales in the range of 0.2–1 s), which extend across much of MT and therefore neurons with very dissimilar functional properties. These observations are consistent with the idea that correlations over short time scales reflect common driving input or direct connectivity between neurons, while those over longer time scales reflect modulation in the gain of larger networks.

### COLUMNAR ORGANIZATION OF MT

Electrophysiological recordings approximately tangential to the cortical surface show smooth changes in direction preference in MT. Nearby neurons must have generally similar direction preference, as multiunit activity is well tuned for direction (McDonald et al., 2014), and recordings with laminar probes inserted approximately perpendicular to the cortical surface also exhibit a preponderance of similar direction preferences along each probe (Solomon et al., 2014). These observations are all consistent with the columnar organization of direction preference in marmoset MT. In addition, staining for myelin in marmoset MT reveals quasi-periodic bands, which may align with the distribution of transcallosal afferents arising in the contralateral area MT (Krubitzer, 1995). Functional correlates of this banding pattern have not yet been identified, and it does not appear to be associated with discontinuities in retinotopy (unlike, for example, the discontinuities associated with cytochrome oxidase stripes in area V2).

### DEVELOPMENT AND PLASTICITY OF MT

The rapid postnatal development of marmosets has been instrumental in allowing studies of cortical maturation and plasticity. Area MT undergoes neurochemical maturation in parallel with V1, and ahead of all other visual areas, suggesting that MT may act as an “anchor point” that guides the maturation of cortical areas (Rosa, 2002; Bourne and Rosa, 2006; Warner et al., 2012; Buckner and Krienen, 2013). Indeed, many of the response properties of MT neurons can develop even when V1 is lesioned in early postnatal life, including normal receptive field topography and short latency responses to visual stimuli. Direction selectivity, however, the characteristic functional feature of neurons in MT, fails to develop in the absence of V1 (Yu et al., 2013). The effects of V1 lesions are age-dependent, as lesions in adults substantially reduce the proportion of responsive neurons in MT, but do not abolish direction selectivity (Rosa et al., 2000); the latter observation is in line with results in the macaque (Rodman et al., 1989).

## THE “MT SATELLITES”: AREAS MST, FST, MTC

As in other primates studied, area MT is neighbored by a complex of areas that have strong interconnections with area MT, and contain relatively high proportions of neurons showing motion selectivity (Krubitzer and Kaas, 1990; Palmer and Rosa, 2006a). These areas might provide complementary or higher stages of motion processing.

### AREA MST

Medial superior temporal area lies anterior to MT, near the tip of the lateral sulcus (Krubitzer and Kaas, 1990). The pattern of visual field representation suggests that this area may be further subdivided, although whether this is warranted remains unclear (Rosa and Elston, 1998). The vast majority of neurons in MST show strong direction selectivity, and have receptive fields predominantly in the peripheral visual field, which are on average larger than those in the corresponding part of area MT. Area MST forms one of the main sources of feedback-type projections to MT (i.e., projections that originate primarily from infragranular neurons; Palmer and Rosa, 2006a). As in the macaque (Boussaoud et al., 1990), marmoset MST receives a small but distinct projection from the representation of peripheral vision in V1, as well strong inputs from areas MT and MTC (Palmer and Rosa, 2006b). Other inputs arise in dorsal and medial extrastriate areas that emphasize peripheral vision (DM, DA, 19M, 23V, area prostriata), in FST, in visual association areas in the posterior parietal cortex (primarily LIP and PPM), in the superior temporal polysensory cortex (STP/TPO), in the parahippocampal cortex (TF) and in frontal lobe areas (primarily 8aV and 8aD; Palmer and Rosa, 2006b). Finally, MST has sparse connections with motor and premotor areas (Burman et al., 2014a,b), and to caudal auditory association areas (Palmer and Rosa, 2006b), suggesting roles in visuomotor and polysensory integration.

### AREA FST

Another major source of feedback-type connections to marmoset MT is area FST (Krubitzer and Kaas, 1990; Palmer and Rosa, 2006a,b). Unlike MST, FST lacks the marked emphasis on peripheral vision, and fewer neurons show clear direction selectivity (Rosa and Elston, 1998). Other than the major projection to MT, FST also projects to other visual areas (e.g., V2 and DM; Jeffs et al., 2009; Rosa et al., 2009) and frontal area 8aV (Reser et al., 2013). FST may be a major node of integration between the dorsal and ventral streams of processing (Rosa and Elston, 1998).

### AREA MTC

The MTC area forms a topographically organized, horseshoe-shaped ring around much of MT (**Figures 1** and **Figures 5**), and may be related to the “V4t” area described in the macaque (Gattass et al., 1988). Area MTC is a major source of input to MT, but unlike in FST and MST these connections originate in equal proportion in the supragranular and infragranular layers, suggesting that they are better thought of as lateral, rather than feedforward or feedback, connections (Palmer and Rosa, 2006a). Receptive fields are, on average, slightly larger than those in MT (Rosa and Elston, 1998), and only half of the neurons show clear direction selectivity. By comparison with MT, MTC receives input from a wider variety of frontal areas, including subdivisions of the ventrolateral and orbital frontal cortices, as well as oculomotor centers (Burman et al., 2006).

## OCCIPITOPARIETAL AND CAUDAL PARIETAL AREAS

Anterior to DM lie areas of cortex that are cytoarchitecturally intermediate between the “classical” (area 19-type) extrastriate cortex and the posterior parietal (areas 5 and 7-type) cortex. This region of cortex is likely to be a site of visuomotor integration, and includes areas whose likely counterparts in macaque are buried deep in the annectant gyrus and parietooccipital sulcus. Receptive field topography and response properties suggest at least two subregions: area DA, which contains neurons with clear visual receptive fields, and a medial region (PPM) where visual responses are harder to obtain in anesthetized preparations. Both DA and PPM are heavily interconnected with areas DM, MT, and MST, suggesting that they are part of the dorsal stream of visual processing (Palmer and Rosa, 2006a,b; Burman et al., 2008; Rosa et al., 2009; Jeffs et al., 2013). In addition, they have reciprocal interconnections with frontal motor, premotor, and oculomotor areas (Burman et al., 2006, 2008; Reser et al., 2013).

### AREA DA

Area DA (or one of its subdivisions) is likely to be homologous to macaque area V3a. DA is topographically organized and includes neurons with relatively large receptive fields, which grow from ∼5° diameter in the central representation to ∼30° in the periphery (Rosa and Schmid, 1995; Jeffs et al., 2013). The topographic organization is complex, with some evidence for two visuotopic maps (Rosa and Schmid, 1995).

### AREA PPM

Based on its connectivity and location, area PPM is likely to correspond to macaque area V6a (Burman et al., 2008; Paxinos et al., 2012). Many neurons in area PPM do not respond to simple visual stimuli under anesthesia (Rosa and Schmid, 1995; Rosa et al., 2009); among those that do respond, receptive fields are very large and diffuse. Area PPM is adjoined anteriorly by putative homologs of macaque area PEC (caudal subdivision of cytoarchitectural area PE) and PGM (medial subdivision of cytoarchitectural area PG), which form other connectional nexuses between visual areas and the premotor centers of the frontal lobe (Burman et al., 2008; Reser et al., 2013).

## POSTERIOR PARIETAL CORTEX

This region comprises a series of architecturally distinct fields (Rosa et al., 2009; Paxinos et al., 2012; Reser et al., 2013) but knowledge of their functional properties and precise boundaries requires further study, preferably in awake-behaving preparations. Among the best characterized subdivisions is a putative homolog of area LIP, which as in the macaque forms strong projections to MT and to the frontal eye fields, and is heavily myelinated in comparison with other “intraparietal” areas (Rosa et al., 2009; Reser et al., 2013). Likely homologs of the medial and ventral intraparietal areas (MIP and VIP), of medial parietal area PGM (medial subdivision of PG) and of ventral parietal areas OPt, PG, PFG, and PF have also been suggested, based on cyto- and myeloarchitecture (Rosa et al., 2009; Paxinos et al., 2012). Large visual receptive fields have been recorded in the likely homologs of OPt and LIP/VIP (Rosa and Tweedale, 2000; Rosa et al., 2005). As in other primates, large lesions that include multiple subdivisions of the posterior parietal cortex result in contralateral neglect (Marshall et al., 2002).

## VENTRAL STREAM AREAS

Our knowledge of the ventral stream areas of the marmoset is still in its infancy. The location and topographic organization of the likely homolog of area V4 (VLA) have been mapped in detail. In addition, area ITc has been defined, which bears strong resemblance to macaque area TEO in terms of location, cytoarchitecture, receptive field size and topography (Rosa and Tweedale, 2000). Both areas are preferentially activated by complex visual stimuli (Ciuchta et al., 2013). Areas VLA and ITc both send feedback-type connections to the central representations of areas V1 (Rosa and Tweedale, 2000; Lyon and Kaas, 2001) and V2 (Jeffs et al., 2013). Whereas VLA also sends topographically organized connections to dorsal stream areas DM and MT, projections from ITc to dorsal stream cortical areas appear to be very sparse (Palmer and Rosa, 2006a; Rosa et al., 2009; Jeffs et al., 2013).

The rostral subdivisions of the inferior temporal cortex of the marmoset are known primarily from histological analyses (Burman et al., 2011; Paxinos et al., 2012), which suggest a close resemblance with these regions in the macaque (**Figure 1**). Dorsal (ITd) and ventral (ITv) cytoarchitectural areas are currently recognized, but these are likely to include multiple functional subdivisions. Although full reports of the response properties of neurons in the different subdivisions of the inferior temporal cortex have yet to appear, there have been preliminary reports of subregions containing face-selective cells (Tamura and Fujita, 2007; Hung et al., 2013). In addition, it has been established that lesions of the marmoset inferior temporal cortex result in deficits in visual object discrimination (Ridley et al., 2001).

## FRONTAL ASSOCIATION AREAS

The frontal eye field of the marmoset has been identified based on both physiological (Blum et al., 1982) and cytoarchitectural (Burman et al., 2006; Burman and Rosa, 2009) criteria. As in the macaque, the frontal eye field is approximately coincident with cytoarchitectural area 8aV, although it may extend further ventrally to include area 45 (Reser et al., 2013). Most (if not all) extrastriate areas have connections with the frontal eye field, but projections from V1 are absent. There is some topography in the connections between extrastriate cortex and the frontal lobe, with the anterior part of area 8aV receiving connections from neurons with receptive fields in peripheral vision, and the posterior part receiving connections from those representing central vision (Reser et al., 2013). Other areas of the frontal lobe, including areas 8aD and 8C, and the rostral premotor cortex, receive sparse projections from extrastriate cortex. These projections originate primarily from dorsal stream visual areas such as MST, FST, LIP, and 19M (Reser et al., 2013; Burman et al., 2014b), and may have a role in the visual guidance of motor activity.

## SUMMARY AND CONCLUSION

We set out to establish the current state of knowledge on the visual system of the marmoset. The most studied stages of visual processing, in the marmoset as in the macaque, are the retina, LGN, and cortical areas V1, V2, and MT. We have shown that in marmosets the corpus of knowledge available for these areas is now solid enough to allow high-level experimental design that exploits the advantages that marmoset monkeys may provide. Among these areas there appear to be no substantive functional or anatomical properties that distinguish marmosets from macaques, provided that the smaller eye and polymorphic color vision of the former are taken into account. Indeed, the simpler geometry of the thalamus and cortex in the marmoset has already allowed sharper understanding of the relationship between structure and function in LGN and MT.

The last decade has seen rapid progress in the establishment of robust protocols for electrophysiology in anesthetized preparations (Yu and Rosa, 2010), structural MRI (Bock et al., 2009), functional MRI (Belcher et al., 2013; Liu et al., 2013), optical imaging (Valverde Salzmann et al., 2012), and behavioral study of eye movements (Mitchell et al., 2014), among other important developments. Although the full extent to which marmosets can be trained in visual tasks has yet to be established, there are indications that, given appropriate training, they can offer reliable performance in tests requiring relatively complex cognitive processes (Dias et al., 1996; Spinelli et al., 2004; Rygula et al., 2010; Tokuno and Tanaka, 2011). In addition, we have not touched on one of the strong advantages of the marmoset in developing primate models of normal vision and visual dysfunction – the potential for genetic modification (Sasaki et al., 2009). The precise functional organization of visual cortex, combined with the availability of embryonic tissue, rapid postnatal maturation and potential for genetic manipulation, mean that the marmoset may provide a tractable model for the study of the detailed molecular events that guide development of the primate cerebral cortex (Bourne et al., 2005; Teo et al., 2012; Goldshmit et al., 2014; Homman-Ludiye and Bourne, 2014). For these reasons we suggest that the marmoset is a sufficient model of primate vision.

Away from the areas of intense research interest mentioned above, our understanding of the visual system in marmosets, macaques, and humans remains incomplete. In the case of most other extrastriate areas, as well as visual association areas of the parietal, temporal and frontal lobes, further comparative work is required to solidify knowledge regarding homologies between primate species. We believe, moreover, that the marmoset will be a necessary model for understanding the roles of these areas in vision. This is because most of these areas appear to be particular specializations of the primate cortex, and in the marmoset these areas lie exposed on the cortical surface, amenable to cellular-resolution imaging and large-scale electrophysiological recording. We invite the reader to imagine what may be learnt by measuring population activity simultaneously from all visual areas between V1 and MST, together with parietal areas such as LIP, during active vision in normal adults. This is already technically achievable. Now imagine what may be learnt about detecting and treating the visual deficits that accompany normal aging and retinal disease, or understanding the brain plasticity that follows stroke.

Finally, the inter-individual organization of marmoset groups has many parallels to human societies, including strong family and peer interactions during development. Marmosets may provide a natural model of visual communication and its development (e.g., Kawai et al., 2014). In conjunction with recently developed techniques for genetic manipulation, which will soon allow transgenic lines with expression of genes known to represent risk factors (Kishi et al., 2014), marmosets will likely become particularly important in understanding the physiological, anatomical, and cognitive correlates of mental disorders, such as schizophrenia and autism.

## AUTHOR CONTRIBUTIONS

Samuel G. Solomon and Marcello G. P. Rosa conceived and wrote this review.

## Conflict of Interest Statement

The authors declare that the research was conducted in the absence of any commercial or financial relationships that could be construed as a potential conflict of interest.
